# Service users and maternity care professionals’ perspectives of apps during pregnancy and childbirth: A narrative review

**DOI:** 10.18332/ejm/225331

**Published:** 2026-11-09

**Authors:** Denise O'Brien, Barbara Coughlan, Jean Doherty, Lorraine Carroll, Ann M. Hogan, Orla Mongan, Mary Curtin, Jennifer Dempsey, Caroline E. Fox, Helen McLoughin, Ann Nwagwu, Lucille Sheehy, Sinead Thompson

**Affiliations:** 1 UCD School of Nursing, Midwifery and Health Systems, University College Dublin, Dublin, Ireland; 2 National Maternity Hospital, Dublin, Ireland; 3 St. Luke’s General Hospital Kilkenny, Ireland; 4 Wexford Maternity Hospital, Wexford, Ireland; 5 Maternity Department, Midland Regional Hospital Mullingar, Ireland; 6 School of Nursing and Midwifery, Trinity College Dublin, Dublin, Ireland

**Keywords:** pregnancy, information, digital Health, apps, informed

## Abstract

There is a strong emphasis on the need for early informational support for pregnant women to optimize the health and development of mothers and newborns. Over the last ten years, smartphones have emerged as the primary means of accessing information. Previous literature on the design, content, and application of pregnancy apps highlights that although there is a high number of apps targeting pregnancy, quality and usability are highly varied, as are the approaches and outcome measures used to evaluate their impact. Because of the inconsistencies reported, the aim of this narrative review is to synthesize and interpret existing qualitative evidence regarding the contribution of apps providing information during childbirth. Five databases, PubMed, Embase, CINAHL, PsycINFO, and Google Scholar, were searched for peer-reviewed literature published between 2010 and 2025. Qualitative and mixed methods studies written in English reporting service users' and maternity care professionals’ perceptions of the use of apps were included. Two reviewers screened and completed data extraction. Narrative data synthesis was completed using thematic analysis. Thirty-nine studies that met the inclusion criteria were included in the review, thematic analysis was conducted, and four major themes were identified: women want apps that are simple, interactive, and visually appealing, that provide trustworthy information developed collaboratively with professionals. Barriers to accessing pregnancy apps still exist, particularly for women from lower socioeconomic groups. There is a need for further research as many apps currently used by pregnant women are difficult to access, not always developed collaboratively with women, and frequently not evaluated for their outcomes and impact.

## INTRODUCTION

Delivery of evidence-based information in a fair and fast format for all is key to ensuring women are well informed for pregnancy, labor, and the postnatal period. In Ireland, the Department of Health published the Digital for Care: A Digital Health Framework for Ireland 2024–2030^1^ in 2024, as a roadmap to digitally transform health services in Ireland. The vision is to create better health outcomes through a digitally enabled environment, including the development of a health app giving service users access to their own health information. The need for a digitally enabled environment is reflected across maternity care policy internationally, and to adapt to this trend, maternity healthcare professionals have incorporated platforms such as specified websites and digital apps for antenatal education. Mobile devices have the potential to reach multiple people at the same time, thus have a far-reaching value in healthcare, with uses for diagnostics, health management, and telemedicine. A significantly increased focus on the use of digital health technologies as a tool for mental health during pregnancy and high-risk pregnancy specifically was highlighted by a recent scoping review by Mohamed et al.^2^.

According to Dalton et al.^3^, increased use of these platforms may potentially improve women’s understanding and knowledge of personal health and healthcare options and enhance their ability to make the most appropriate choices for pregnancy. Although a substantial amount of health-related information is available online, the volume of content often makes it challenging for women to navigate effectively. Consequently, there is a clear need for accessible, evidence-based information sources and reliable digital applications^3^. To enhance access to information, digital and app-based interventions, as well as educational platforms developed by qualified healthcare professionals with appropriate expertise, have been advocated^4^. There has been a growing interest in the capability of smartphone applications (‘apps’) to promote health, encourage behavior change, and enhance the service users’ experience. Whilst there are strong advocates for the use of mobile technology in childbirth education, a scoping review by Auxier et al.^5^ reveals that evidence about their impact on pregnancy wellbeing and behavioral changes following app interaction is lacking.

A systematic review evaluating apps for pregnant women highlighted that more research is needed, as many apps lacked evaluative research^6^. The evidence that does exist of apps’ positive contribution as a source of education is promising. That said, in terms of the behavior-changing impact, the findings are divergent. For example, Foster et al.^7^ found that most of the 14 women who piloted an mHealth app developed in Atlanta specifically for African American women at high risk for adverse outcomes, perceived the app as helpful; however, logistical challenges to app management were identified as a significant limitation, with minimal service user behavioral change noted. Similar findings are reported by Hall et al.^8^ who conducted ten focus group meetings with 26 mothers to explore their use of digital technologies during the transition to parenthood in Canada, reporting mixed findings. From a positive perspective, digital technologies enabled and facilitated mothers’ engagement in behaviors of self-surveillance, basing their behaviors and decision-making on information they gathered online. Cultural and socioeconomic factors were highlighted as factors that hindered engagement and limited impact in terms of outcomes. In general, Hall et al.^8^ found that women seek to use pregnancy and childbirth health apps in a ‘mix and match’ approach, along with a wide range of other sources, including friends, family, and healthcare professionals. Because of the inconsistencies reported, the aim of this narrative review was to synthesize and interpret existing qualitative evidence regarding the contribution apps have in providing information to women in pregnancy, childbirth, and the postnatal period, and to examine their contribution and impact on women.

A narrative literature review provides an in-depth analytical assessment of contemporary knowledge on a topic and is useful to provide context for further research. A narrative review was chosen to give a detailed overview of qualitative findings of service users and maternity care professionals’ perspectives on the current contribution of apps as an educational platform. Narrative reviews are appreciated for offering in-depth insights into a research topic by synthesizing, critiquing, and interpreting existing literature^9^. The authors began this narrative review by conducting a review of the literature. The preferred reporting system for systematic review (PRISMA) recommendations was followed for this review. An experienced university librarian assisted in identifying search terms. The focus of the review was clearly defined, and the inclusion and exclusion criteria, as well as assessing quality and eligibility, were agreed upon.

A search of the literature was undertaken using five databases, PubMed, Embase, CINAHL, PsychINFO and Google Scholar. These databases were searched in consultation with a subject librarian and were chosen to capture an extensive range of literature. The mesh terms ‘Apps’, ‘digital health’, ‘mHealth’, ‘pregnancy’, ‘new mother’, ‘childbirth’, and ‘maternity care professional’ were included to search all studies published in English between 2010 and 2025. They were combined with the Boolean operators AND and OR (Supplementary file). The focus was on maternity care professionals and service users’ perceptions of apps as an informational resource during pregnancy and childbirth.

Included studies were quality appraised using the Critical Appraisal Skills Program’s (CASP) Qualitative Studies Checklist and the Mixed-Methods Appraisal Tool (MMAT), where relevant. Identified studies were appraised by two reviewers (DOB, AN). Data were extracted in Covidence (AN, DOB). Any conflicts were resolved by a third author (ST). Thematic analysis was used; we adapted the six-step process of Braun and Clarke^10^ methodology to synthesize qualitative and mixed-method findings. Initially commonalities and differences were coded, and key themes were identified and agreed upon (DOB, ST).

## COMMENTARY

Within our narrative review, 10856 studies were identified, reduced to 10650 after 206 duplicates were removed. Following title and abstract screening, 168 studies were considered potentially relevant and were subsequently assessed through full-text evaluation. Thirty-nine studies were eligible for inclusion in the review (Figure 1).

**Figure 1:**
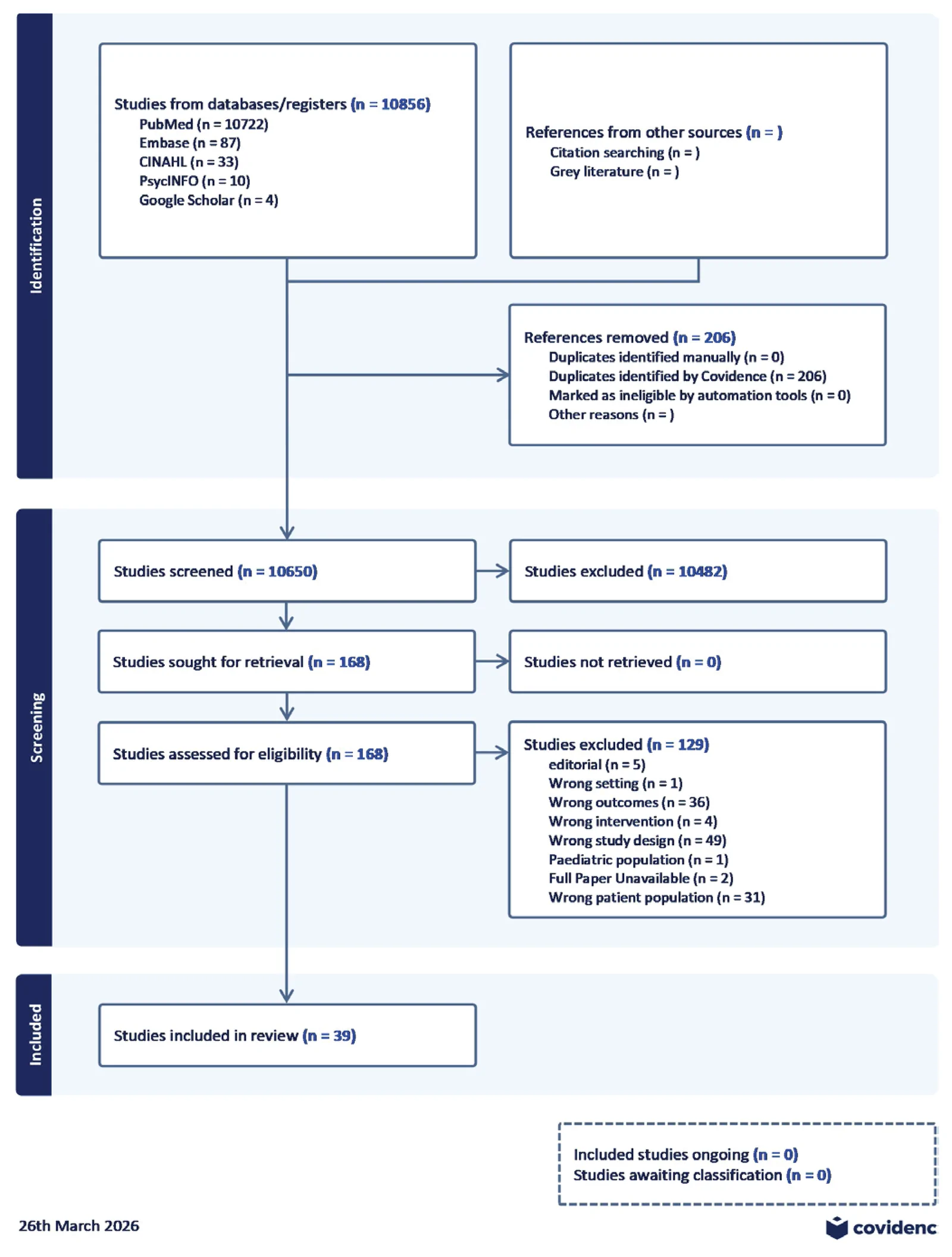
PRISMA flow chart of selection of studies

Most of the studies employed a mixed-methods approach, most publications were from the USA (n=7), the United Kingdom (n=6), and Australia (n=6) (Figure 2).

**Figure 2:**
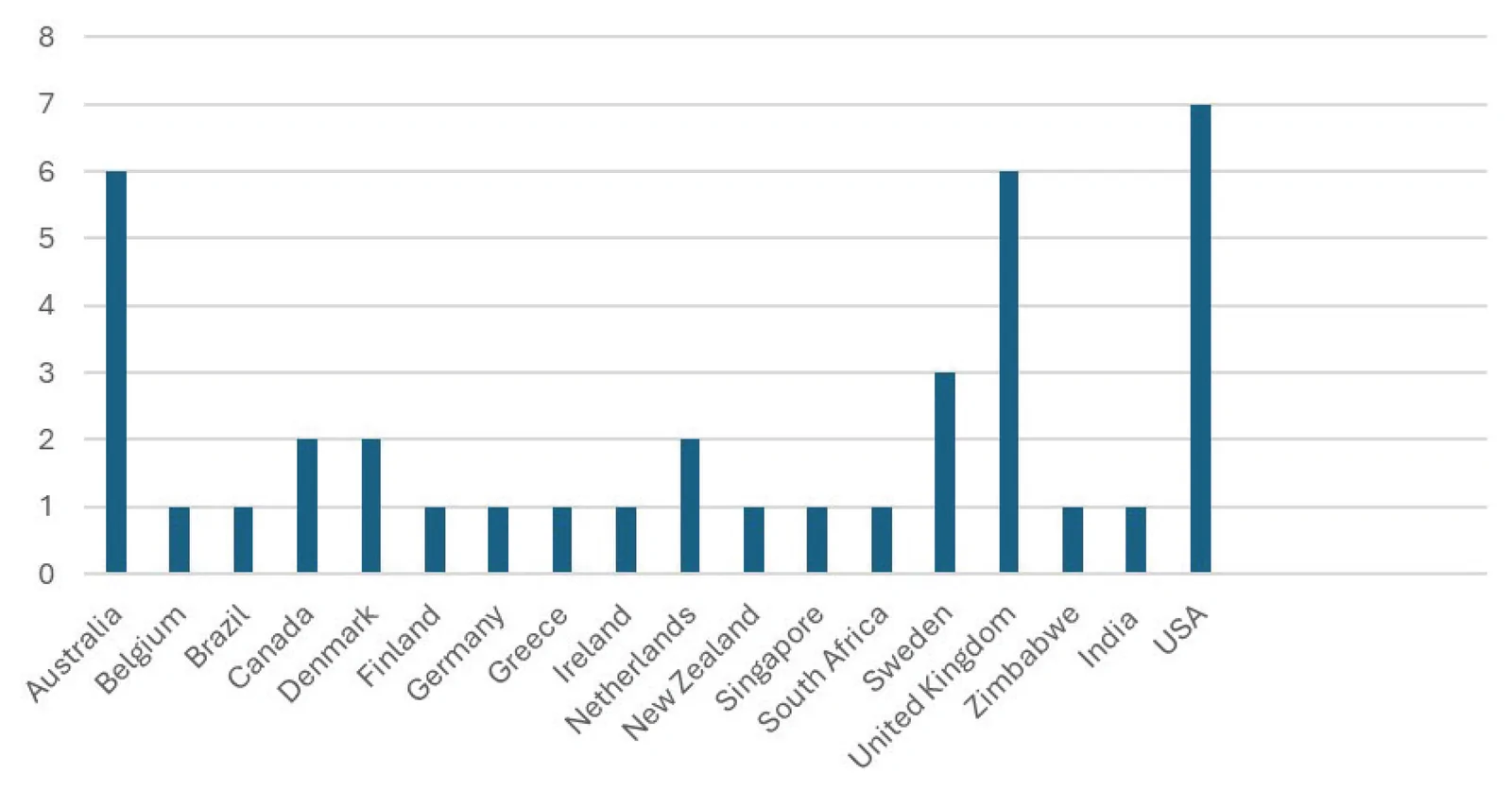
Publications by country

The narrative synthesis details the findings from the original studies, highlighting evident similarities, differences, and any potential contradictions. Four themes were identified that reflected perspectives about apps during pregnancy and childbirth: women want apps that are simple, straightforward, interactive, and visually appealing; collaborative women-centered development works best; trustworthy information is key; and barriers still exist.

### Women want apps that are simple, straightforward, interactive, and visually appealing

This theme presents what women want from apps so that they can be considered women-centered. What is evident is that women want apps that are simple to use. Bailey et al.^4^ found that formatting and presentation of information were key to the success of app usage during pregnancy. The Baby Buddy App was co-designed with mothers to support and guide women through pregnancy (antenatal) and the first six months of their child’s life (postnatal). Bailey et al.^4^ found that women liked the format in which the information was presented using the Baby Buddy App, which was easily accessible and could also be shared with friends and family. Ease of access is a common trend in terms of importance when service users evaluate apps. In a qualitative review of user experiences with ten different pregnancy apps, Hossain et al.^11^ highlighted that service users valued detailed, relevant information that was easy to access. Ease of access and functionality were also highlighted as crucial factors to the usage and success of pregnancy apps by Fritzon et al.^12^, in Sweden. This need for easily accessible information in a format that women want is a recurring finding. Bogaerts et al.^13^ in Belgium developed and evaluated the INTER-ACT App in Belgium as part of a pregnancy lifestyle coaching module. Both mothers and healthcare providers valued the interactive nature of the app and considered it beneficial in combination with face-to-face coaching. The positive benefits of designing apps to support patient engagement were also reported by Goetz et al.^14^, in Germany, who developed an app to promote healthy lifestyle choices. Women stressed the need for a user-friendly interface in apps. During a usability assessment of an app developed for diabetes during pregnancy in Illinois by Yee et al.^15^, ease of access to visually appealing evidence-based information was identified as a key strength that supported usability. This is a frequent finding from several studies exploring the development of apps for diabetes in pregnancy. Larsen et al.^16^ conducted a mixed-methods study with 42 women in the US to evaluate the feasibility and acceptability of a physical activity intervention for pregnant women with diabetes. Follow-up interviews revealed that participants enjoyed the interactive nature of the app and found it simple and easy to use. Kyoto et al.^17^, in Finland, also conducted interviews with participants following the development of an interactive gestational diabetes prototype app. The findings revealed that participants were generally very positive and wanted simple apps that are interactive and easy to use. Lau et al.^18^, in Singapore, also reported high rates of end-user satisfaction with app usage. Sixteen participants were involved in the development of the app (mHELP), which aimed to provide a healthy lifestyle program among overweight or obese multi-ethnic pregnant women. The general preference of participants was the use of interactive multimedia, including short video scripts, graphs, photos, web-based quizzes, and animation, to make the app highly engaging. So, based on the findings from this narrative review, it is important that information provided within apps is delivered in simple, interactive, and visually appealing formats.

### Trustworthy information is key

A central finding that must be considered for those delivering information is that the usability of an app is contingent on women trusting the information provided. Women want to see that the information provided is authentic and approved by maternity care professionals. For example, women in the Bailey et al.^4^ study, reported feeling reassured that the Baby Buddy App was developed by maternity care professionals, and that they were receiving reliable information. Boe Danbjørg et al.^19^ also found that an app developed to provide support in the early postnatal period following discharge, was a welcome source of information and accepted as a substitute for face-to-face contact at the hospital. Women will seek apps that are reviewed by medical professionals or endorsed by relevant health authorities. Bogaerts et al.^13^ found that women were highly receptive to apps developed by maternity care providers and more likely to engage with them when evaluating an app for weight management during pregnancy. Doherty et al.^20^ developed BrightSelf, an app for the self-report of psychological wellbeing during pregnancy, in London. Women described the app as a safe space for disclosing how they were feeling and were more likely to engage with it because it was developed and managed by maternity care providers, which enhanced its credibility and trustworthiness. Viewing apps as a safe space was also reported by Fritzon et al.^12^ who found that safety and security were important aspects of feedback gained from the app they developed for women in early labor, in Sweden. The need for a secure safe space to access information was also highlighted by Goetz et al.^14^ who found women had a high demand for ‘safe’ information during pregnancy, because they distrusted much of the information on the internet. Therefore, the development of interactive personalized mobile apps was considered necessary to meet their needs for information. Gourounti et al.^21^ explored the views of pregnant women in Greece about the use of the internet as a source of information. Once again, trust was an important issue; women reported preferring to get information from websites of well-known and recognizable organizations. Many of them expressed concerns about the trustworthiness of online sources of information and did not trust information that was not affiliated with a healthcare professional. The need for professional input was also reported by Arbour and Stec^22^, who reviewed available apps for pregnancy in the US, concluding that the most important aspect of initiation and integration of mobile apps into clinical practice is to ensure they are peer reviewed or from appropriate professional sources. The same requirement for trustworthy information from professionals was also reported from Ireland. Exploring the acceptability of the mixed lifestyle exercise and nutrition during pregnancy intervention (PEARS) smartphone app^23^ revealed that participants appreciated that the PEARS app contained reliable information, approved by healthcare professionals. The benefits of providing information from trustworthy sources were highlighted in a qualitative study by Sandborg et al.^24^ with 19 women within the ‘Healthy Moms Trial’ in Sweden. The findings illustrated that a trustworthy health and pregnancy app that is easy to use can inspire a healthy lifestyle during pregnancy. Similar findings were reported^25^ from Singapore exploring the views of new parents about the content and delivery of a mobile health (mHealth) app-based postnatal educational program. Seventeen parents took part in interviews, and the key message was that the facilitator of the featured communication platform, a midwife, provided trustworthy advice. A sense of belonging to a virtual community beyond the hospital gave parents the confidence that they were not alone and were supported by other parents and healthcare professionals. For those designing apps for pregnancy, it is clear that women want information they can trust, verified by a recognized healthcare professional.

### Collaborative women-centered development works best

A theme that emerged from this review was that collaborative development of online support between service users and healthcare professionals was seen to lead to greater engagement by service users. The key strength of collaboration was the active involvement of end users from the start of the design process, allowing for a clear understanding from the outset of users’ experiences and requirements for online information and support. For example, Van Beukering et al.^26^ explored the usability and usefulness of a mobile health app for pregnancy-related work advice in the Netherlands and found clear benefits to including service users in design to improve usability. Engaging with women reduces costly redesigns, fosters deep user trust, and ensures the app provides clinically accurate and culturally sensitive support. Rather than making assumptions, we need to find out the format women want to receive information, as Mitilello et al.^27^ identified, women describe themselves as visual learners and concluded that women need to be involved from the onset so that technology can be developed to meet their individual preferences, including what they see as priorities and relevant. Evidence of the strength of co-designing with women was found by Moraes Carrilho et al.^28^ who involved users in Brazil in the prototyping phase of an app development to identify problems, and based on recommendations from women, the design team redesigned and made improvements to the app. How service users can improve design was also highlighted by Brunelli et al.^29^ who adopted a co-design approach engaging with 94 pregnant women and 69 partners to develop an app to support the first 1000 days of life. Participants generally agreed with the proposed content but expressed low interest in certain app functionalities and features. Women generally expressed greater information needs than men. The findings highlight that the engagement of end-users may be useful for designers and technology providers to implement mHealth solutions by designing apps that offer intuitive navigation to reliable information, meeting the needs and preferences of end-users. Bailey et al.^4^ also used a participatory approach, utilizing Appreciative Inquiry (AI) to engage with women in Australia to understand when, why, and how first-time mothers use the Baby Buddy app and the perceived benefits and challenges. Engaging with women was considered to contribute to positive evaluations, and service users considered the information reliable due to its association with HCPs in the video clips and written content. Christiansen et al.^30^ also used a participatory design approach and the behavior change wheel to develop an app to support a healthy postpartum lifestyle through behavioral change techniques. The app was developed and designed for Danish postpartum women, who were involved from the onset. This was deemed key to the success of the project, reflecting mothers’ requests in a format that they wanted. Relevance can also be enhanced by adopting a multidisciplinary approach. Barber et al.^31^ developed and piloted an app with 88 women in New Zealand called ‘Positive Pregnancy’ through collaboration with midwives, antenatal educators, pregnant women, and clinical and community psychologists. The app was developed based on findings and submissions from a survey seeking content recommendations. The results of the piloted prototype app revealed that a large group of users were neutral or mixed in their satisfaction with the app. Recommendations were for less wordy information, and more graphics, navigation, and screen display and organization of information were deemed important. These recommendations were used to make changes to the app, and the app was provided free of charge to women in New Zealand. What became apparent from collaborating with women is that they want visual interactive learning and not dense sections of information^31^.

Collaboration with service users is key; equally important is collaboration with healthcare providers. Wit et al.^32^ stress the importance of involving midwives early in the development of apps, which may facilitate better alignment with the needs and preferences of service users. White et al.^33^, in Australia, also advocate that midwives need to be involved in the development of m-health interventions for them to be acceptable and tailored to their needs in a rural and remote context. Sandborg et al.^24^ also adopted a collaborative approach with midwives during the development of a user-friendly, evidence-based, fit-for-purpose app. The authors report that involving midwives has guaranteed that the content was designed to meet specific user needs in practice. Co-designing and collaboration with service users and healthcare professionals ensure that the final digital tool addresses real-world maternal needs rather than developer assumptions, leading to highly relevant, supportive, and trusted pregnancy apps.

### Barriers to app usage

Although significant improvements have been made relating to access to large volumes of pregnancy-related information, inaccurate assumptions are often made about people’s ability to access and use mobile technology to access information. This review has found that barriers do still exist. Many apps contain inaccurate information or contradictory advice, resulting in a lack of credibility. Users also worry about the security of their health data and lack of transparency; apps are often highly monetized and feature excessive ads. In addition, excessive push notifications and high battery/memory usage are cited as significant barriers. Digital exclusion exists, as barriers disproportionately affect vulnerable demographics. This review has revealed that language barriers and lack of access to a reliable smartphone are significant hurdles, particularly among asylum-seeking populations and ethnic minorities. This is most evident amongst women with low health literacy, as highlighted by Dalton et al.^3^. People from lower socioeconomic groups and low health literacy are more likely to have poor outcomes. Education is therefore important, and a concern of the authors is that women from lower socioeconomic groups are less likely to engage with pregnancy apps. Dalton et al.^3^ found, when conducting the Health-e Babies App in Australia, that multiple challenges existed that prevented access to apps such as financial constraint, technological ability, and mental health issues. Dalton et al.^3^ focused on health outcomes in pregnant women from socially disadvantaged populations. Of note, despite social disadvantage, all women had a mobile phone, 50% of the group (n=151) used apps daily, and wanted accessible pregnancy-related information. Subsequently, the authors developed an app specific to the needs of the population of women. However, only 24% of the group (n=60) used the app and completed the study. The qualitative findings revealed that the additional charges associated with extra data and access to external websites limited app use. Similar difficulties were reported in a narrative review of MomConnect, an mHealth resource offering antenatal and maternal health information launched in South Africa to improve maternal health^34^. Findings from the review found that MomConnect has a large user group with over half a million users and overwhelmingly positive feedback from women. However, high maintenance costs, restrictions on using the Unstructured Supplementary Service Data (USSD) system, which is prone to network issues and a lack of coverage, were cited as significant barriers. Lovell and Haris^35^ evaluated women’s use of mobile apps in labor in the United Kingdom. Of the 749 respondents, 129 responded to the free-text question. The responses were analyzed using an inductive thematic approach, and three main themes were identified: facilitators, barriers, and desires. Negative comments about the actual functionality of apps used included heavy battery usage, difficulty understanding how to use them, and apps crashing. Another issue related to what was missing for women was that the available apps did not meet each woman’s need for information.

### Implications

This narrative review adds to the current understanding of how apps may contribute to women’s informational needs during experiences of pregnancy, childbirth, and the postnatal period. The review also provides important information about midwives’ perspectives on the contribution of apps and how they should be developed. Information accessed from high-quality, regularly updated apps that reflect current evidence will benefit maternity care professionals and women. Mobile health information through apps presents an opportunity to reach a larger number of women. The evidence from this review reveals that pregnancy apps are an extremely popular medium for pregnant women and are valued by midwives. Women actively use apps to search for pregnancy health-related information, discuss issues with peers, and seek advice from professionals to guide their pregnancy decision-making^22^. For this reason, apps have a secure place in future maternity care policy and practice. Because of the difficulties highlighted in this review in terms of the quality of information often reported as conflicting or inaccurate, it is important that apps are regularly reviewed and content updated. It is also important that maternity care professionals engage at the onset with service users and ascertain what they want in any proposed app that proposes to provide informational support and advice during pregnancy. Although collaborative development is important, of equal importance is the evaluation of impact and outcomes. A clear risk with the use of apps during pregnancy is that many apps lack expert quality assurance, as very few are formally evaluated, and lack research^6^. Although globally millions of pregnant women are using apps daily, very few outcomes are reported, and very little analysis is available confirming that app interventions are yielding their desired results and objectives. In terms of the behavior-changing impact, a significant number of apps on the market lack dedicated behavioral change techniques, demonstrating low scientific rigor. This review reveals that very little evidence exists that offers an evaluation of apps providing tailored goal achievements based on clinical recommendations. There is, however, evidence that women who use specific apps alongside routine care, often will self-monitor their behavior with positive results. These findings have implications for policymakers, particularly as there is increased focus on the benefits of digital health.

Equally important is recognition that socioeconomically disadvantaged women and migrant minority women face difficulties engaging with health professionals’ education apps^36^. It is imperative that these factors are considered if mobile phone apps are to be considered a viable medium of health communication for disadvantaged populations. The findings reveal that app developers should design apps that offer robust offline functionality to counteract the high costs associated with highly interactive apps that require a lot of data. In addition, considerations for providing physical tablet lending programs at local clinics could bridge the gap for marginalized groups. It is important that research findings are reviewed by policymakers prior to implementing changes to the provision of information from a national perspective. Findings reveal that there is a need for more qualitative research exploring how effective apps are at meeting the needs of childbearing women in terms of changing health behaviors and long-term impact. It is also important that midwives and maternity care professionals are included from the onset, to ensure apps are developed that are reflective of current evidence-based practice.

### Limitations

This review is limited in that it is restricted to the English language, and there is potential for selection bias. A significant limitation is that, generally, the findings relate to the perspectives of service users from high-income countries. Lack of universal coverage is an important limitation in many apps. Our findings highlighted that many migrant and lower socioeconomic groups do not have access to funds for smartphones, meaning many apps are not accessible to some of the most vulnerable women.

## CONCLUSION

The evidence supporting pregnant women’s attitudes towards using mobile phones to seek information and self-management services via app technology is significant. That said, there is a need for improvements and further research. Based on the findings of this narrative review, the following recommendations are proposed to improve the value and impact of apps for women during pregnancy. Overall, pregnancy, childbirth, and postnatal information apps should provide clear, practical, interactive information that has a simple design and is easy to navigate. To increase usage, it is recommended to involve end users from the outset in the development of apps. Moreover, it is essential to provide apps without cost from a recognized source (such as the relevant national Department of Health or Health Service). Further research is needed to ascertain the impact of apps and digital forms of education to evaluate them for impact and effectiveness for women during pregnancy and childbirth.

## Supplementary Material



## Data Availability

The data supporting this research can be found in the Supplementary file.
